# BRET Analysis of GPCR Dimers in Neurons and Non-Neuronal Cells: Evidence for Inactive, Agonist, and Constitutive Conformations

**DOI:** 10.3390/ijms221910638

**Published:** 2021-09-30

**Authors:** Chayma El Khamlichi, Laetitia Cobret, Jean-Michel Arrang, Séverine Morisset-Lopez

**Affiliations:** 1 Centre de Biophysique Moléculaire, CNRS UPR 4301, Université d’Orléans, Rue Charles Sadron, CEDEX 2, 45071 Orléans, France; chayma.el-khamlichi@cnrs-orleans.fr (C.E.K.); laetitia.cobret@cnrs-orleans.fr (L.C.); 2 Centre de Psychiatrie et Neurosciences, 2 ter Rue d’Alésia, 75014 Paris, France; jean-michel.arrang@inserm.fr; 3 Institut de Psychiatrie et Neurosciences de Paris, UMR_S1266 INSERM, Université Paris Descartes, 102 Rue de la Santé, 75014 Paris, France

**Keywords:** BRET, brain, constitutive activity, dimerization, GPCR, H_3_R

## Abstract

G-protein-coupled receptors (GPCRs) are dimeric proteins, but the functional consequences of the process are still debated. Active GPCR conformations are promoted either by agonists or constitutive activity. Inverse agonists decrease constitutive activity by promoting inactive conformations. The histamine H_3_ receptor (H_3_R) is the target of choice for the study of GPCRs because it displays high constitutive activity. Here, we study the dimerization of recombinant and brain H_3_R and explore the effects of H_3_R ligands of different intrinsic efficacy on dimerization. Co-immunoprecipitations and Western blots showed that H_3_R dimers co-exist with monomers in transfected HEK 293 cells and in rodent brains. Bioluminescence energy transfer (BRET) analysis confirmed the existence of spontaneous H_3_R dimers, not only in living HEK 293 cells but also in transfected cortical neurons. In both cells, agonists and constitutive activity of the H_3_R decreased BRET signals, whereas inverse agonists and GTPγS, which promote inactive conformations, increased BRET signals. These findings show the existence of spontaneous H_3_R dimers not only in heterologous systems but also in native tissues, which are able to adopt a number of allosteric conformations, from more inactive to more active states.

## 1. Introduction

A large body of biochemical and biophysical evidence indicates that G-protein-coupled receptors (GPCRs) form dimers not only in heterologous systems but also in native tissues [1,2,3,4,5,6]. The observation that GPCRs can form dimers or larger oligomers has led to intense research to study the functional and physiological relevance of such complex formations [7,8,9,10,11].

Although some studies have suggested that agonists can regulate dimers by either promoting or inhibiting their formation [12,13,14,15], a general consensus tends to conclude that dimerization is a spontaneous process that pre-dates receptor activation [6,16,17,18,19]. In addition, in some studies, ligands do not influence dimerization [20,21,22], whereas, in others, they induce important conformational changes in dimers, leading to allosteric interactions between the two protomers [23,24,25,26,27,28,29].

Active GPCR conformations are not only promoted by agonists but also occur in their absence, leading to constitutive activity. Inverse agonists decrease constitutive activity by promoting inactive conformations. Whether an alteration of this constitutive activity by inverse agonists leads to changes in the amounts and/or conformations of dimers remains unclear. In fact, very few studies have addressed the putative relationship existing between constitutive activity and the dimerization of GPCRs. In cross-linking experiments performed on the dopamine D2 receptor, the homodimer interface in the inverse agonist-bound conformation is consistent with the dimer of the inactive form of rhodopsin [25]. In trafficking experiments on the dopamine D1 receptor, inverse agonists induced a conformational change, which stabilized D1 receptor dimers at the cell surface [28]. It was also suggested that inverse agonist binding to the second protomer of a Class A GPCR dimer enhances signaling, i.e., that constitutive activity of this second protomer inhibits signaling [26]. This assumption is consistent with studies on the Class C mGluR, in which the inactive state of a protomer caused by inverse agonist binding results in more efficient activation of the adjacent protomer [30,31]. 

We identified the histamine H_3_ receptor (H_3_R) as an autoreceptor located on histaminergic nerve endings, controlling histamine synthesis and release in the brain [32,33]. Since then, it has been shown that many H_3_Rs in the brain are, in fact, located in many neuronal populations [34] and play a role in the pathophysiology of various central nervous system diseases [35]. The H_3_R is a target of choice for the molecular study of GPCRs [36]. Both the rat and human H_3_Rs display a high level of constitutive activity [37,38,39]. We also demonstrate a high constitutive activity of cerebral H_3_Rs, thereby bringing evidence of the constitutive activity of native GPCRs in vitro and in vivo [38,40,41]. H_3_R dimerization has been reported in previous studies [42,43]; however, the functional consequence of the process has not yet been explored.

In the present study, we used Western blot, co-immunoprecipitation, and bioluminescence resonance energy transfer (BRET) analyses to further investigate the dimerization of recombinant and brain H_3_Rs, as well as their regulation by agonists and inverse agonists in human embryonic kidney 293 (HEK 293) cells and cultured cortical neurons.

## 2. Results

### 2.1. Biochemical Detection of H_3_R Dimers

**Figure 1:**
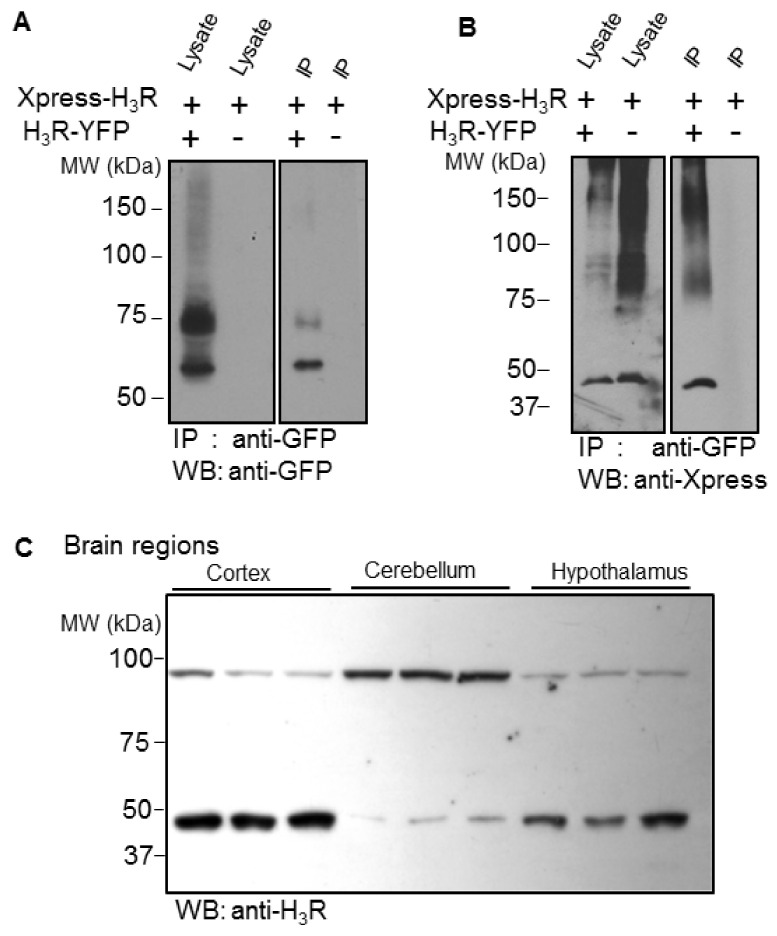
Detection of H_3_R dimers in HEK293 cells and rat brain regions. The Xpress-tagged H_3_ receptor (Xpress-H_3_R) was expressed transiently in HEK293 cells in the presence or absence of H_3_R-YFP. Cell lysates were immunoprecipitated using GFP-Trap agarose beads (**A**,**B**). Lysates or immunoprecipitates (IP) were separated by SDS/PAGE on 8% (**A**) or 10% (**B**) gels. Analysis was performed by Western blot (WB) using a polyclonal anti-GFP antibody (**A**) and a monoclonal anti-Xpress antibody (**B**). Membranes from rat cerebral cortex, cerebellum, and hypothalamus were separated by a NuPAGE 7% Tris-acetate gel system and analyzed by Western blot using the anti-H_3_R antibody (**C**). Similar results were obtained using three different lysates from each brain region.

We first investigated H_3_R dimerization by performing co-immunoprecipitation experiments. HEK293 cells were co-transfected with two different H_3_R constructs, H_3_R-YFP and Xpress-H_3_R, expressing the receptor tagged at its N-terminus with an Xpress epitope. After solubilization, cell lysates were immunoprecipitated with an anti-GFP-antibody and assessed for the presence of H_3_R-YFP with the anti-GFP antibody and for the presence of Xpress-H_3_R with the anti-Xpress antibody. Western blot analysis of the co-immunoprecipitates with the anti-GFP antibody revealed two prominent immunoreactive species at around 75 and 65 kDa (Figure 1A), representing the glycosylated and non-glycosylated monomeric forms of the recombinant H_3_R-YFP (see Figure S1). As shown in Figure 1B, Xpress-H_3_R specifically co-immunoprecipitates with H_3_R-YFP both as monomeric (~45 kDa) and SDS-resistant oligomeric forms (~90 and >120 kDa). We then performed Western blot analysis of membranes from rat brain regions using a specific anti-H_3_R antibody (Figure S2). The labeling of two prominent immunoreactive species was observed with sizes of ~45 and 90 kDa, expected for the monomeric and dimeric forms of the receptor (Figure 1C). 

### 2.2. Detection of Constitutive H_3_R Dimers in Living HEK-293 Cells by BRET

**Figure 2:**
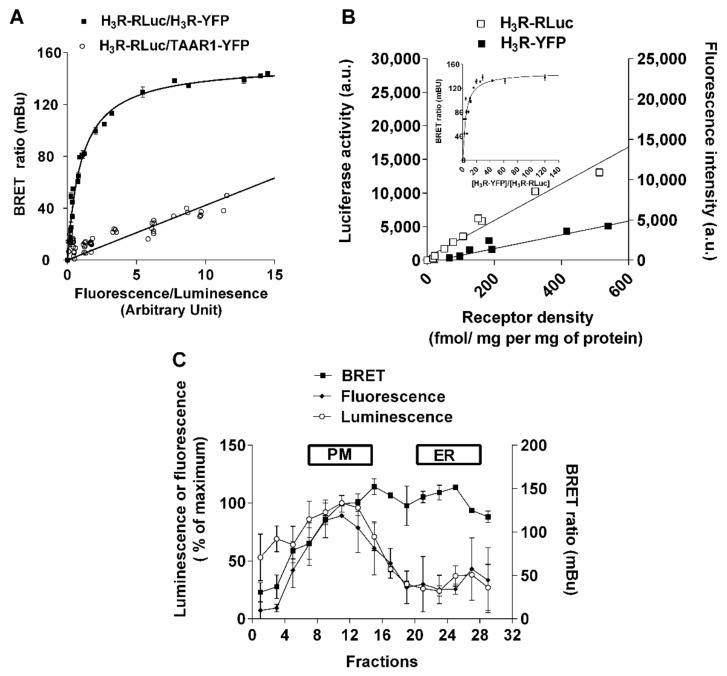
Characterization of H_3_R BRET signals. (**A**) The BRET donor saturation curve of H_3_R-RLuc-H_3_R-YFP (■) is compared with that obtained with a control receptor (○) (TAAR1-YFP). BRET saturation curves were generated by transient transfection of HEK 293 cells with a constant DNA amount of H_3_R-RLuc and increasing quantities of H_3_R-YFP or TAAR1-YFP. BRET, total luminescence, and total fluorescence were measured 48 h after transfection. BRET levels were plotted as a function of the ratio of the expression level of the YFP construct (quantitated by the total fluorescence of the cells) over the RLuc construct (quantitated by the luminescence of the cells) (fluorescence/luminescence). The results are representative of three independent experiments carried out in triplicate. The curve obtained for H_3_R-RLuc-H_3_R-YFP was best fitted with a non-linear regression equation assuming a single binding site (BRETmax = 144 ± 3 mBU), and the curve obtained for H_3_R-RLuc-TAAR1-YFP was best fitted with a linear regression equation. (**B**) To correlate fluorescence and luminescence levels with H_3_ receptor densities, HEK 293 cells were transfected with increasing DNA concentrations of the H_3_R-Rluc or H_3_R-YFP constructs. Forty-eight hours after transfection, luminescence and fluorescence were measured, and H_3_R expression was determined by radioligand binding assay cells that were diluted in HBSS and distributed in 96-well microplates for luminescence or fluorescence measurements at a density of ~100,000 cells per well. To correlate the luminescence and fluorescence values with receptor densities, the specific number of [^125^I]iodoproxyfan binding sites was determined in the same cells. Luminescence (right, Y-axis) and fluorescence (left, Y-axis) were plotted against binding densities, and the linear regression of the data was performed using GraphPad Prism (inset, Figure 2B) as follows: H_3_R-Rluc: y = 26.3x + 467; H_3_R-YFP: y = 8.5x + 258). The BRET saturation curve presented in the inset was generated from the corrected ratio [H_3_R-YFP]/[H_3_R-Rluc], determined by transforming luminescence and fluorescence values measured for each data point into receptor densities using the equations described above. (**C**) Sub-cellular distribution of H_3_R dimers. HEK 293 cells were transfected with H_3_R-RLuc and H_3_R-YFP. Sub-cellular fractions were subjected to fluorescence/luminescence and BRET analysis. Means ± SEM of 6 determinations from 2 separate experiments.

We also studied H_3_R dimerization in intact living HEK-293 cells by BRET analysis with the proper controls required by this approach [44,45,46,47]. The specificity of H_3_R dimerization was shown by the high energy transfer in cells co-expressing H_3_R-RLuc (H_3_R fused to Renilla Luciferase (RLuc)) with H_3_R-YFP (H_3_R fused to Yellow Fluorescent Protein (YFP)), and by the lower energy transfer in cells co-expressing H_3_R-RLuc with TAAR1-YFP. The extent of energy transfer between the RLuc and YFP tags of the H_3_R was plotted as a function of increasing acceptor–donor ratios (Figure 2A). The curve obtained for H_3_R-RLuc-H_3_R-YFP was saturated and best fitted by s non-linear regression equation (BRETmax = 144 ± 3 mBU). When cells were transfected with H_3_R-RLuc, tracemine-associated receptor 1 (TAAR1)-YFP signals were best fitted by linear regression. 

In order to exclude that the dimerization process is due to H_3_R overexpression in the heterologous system, we ensured that H_3_R was expressed at the physiological level in our BRET experiments. For this purpose, the luminescence and fluorescence signals obtained after transfection of different concentrations of H_3_R-RLuc or H_3_R-YFP plasmids were correlated to the receptor density measured in cells by binding experiments (Figure 2B). Then, cells were co-transfected with constant amounts of H_3_R-Rluc (~20 fmol/mg protein) construct and increasing amounts of H_3_R-YFP plasmid. The amount of each receptor species effectively expressed in transfected cells was determined for each condition by correlating luminescence and fluorescence signals with receptor densities (Figure 2B inset). BRET signals increased as a hyperbolic function of the ratio between the BRET acceptor and donor to reach a plateau (maximal energy transfer (BRET_max_)) of ~150 mBU from a ratio of ~15 (Figure 2B). In addition, the formation of constitutive H_3_R dimers did not result from an over-expression of the receptor in transfected cells since BRET signals are independent of H_3_R density (Figure S3). When the ratio H_3_R-YFP/H_3_R-Luc was selected to ensure a BRET_max_ signal, it remained constant over a wide range of H_3_R densities (from 50 fmol/mg protein to 1 pmol/mg protein).

In terms of the dimerization of various GPCRs occurring at early stages of receptor biosynthesis within the endoplasmic reticulum (ER), we further analyzed the distribution of H_3_R dimers by cell-fractionation studies. The distributions of the fluorescent and luminescent receptors both indicated that the vast majority (~80%) of total H_3_Rs were exported from the ER to the plasma membrane (Figure 2C). BRET_max_ values on membrane preparations were slightly higher than in whole living cells, in agreement with various studies indicating that the energy transfer depends on the environment [23]. These maximal BRET values were reached not only at the plasma membrane but also in the ER (Figure 2C), indicating that dimerization of the H_3_R occurs early during its biosynthesis. 

### 2.3. Effects of H_3_R Ligands on BRET in HEK 293 Living Cells

**Figure 3:**
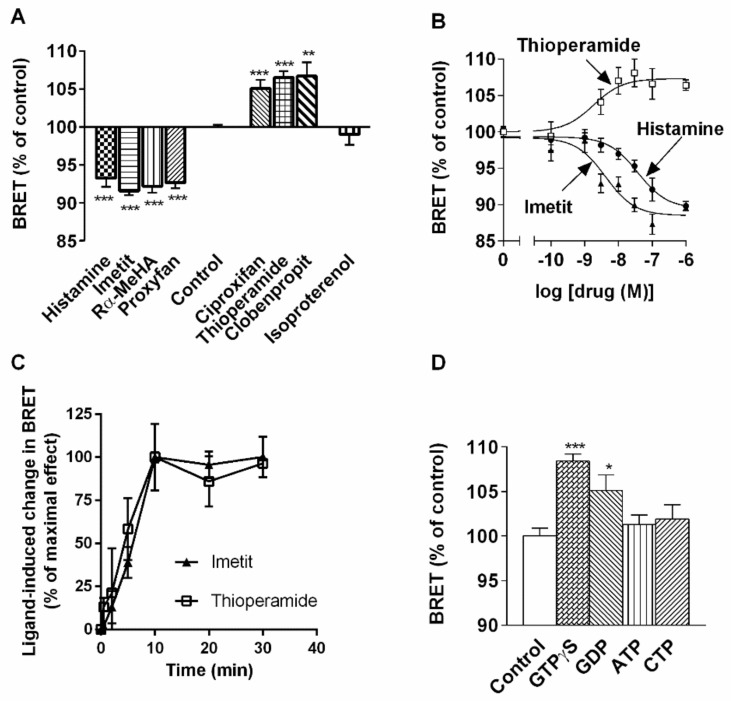
Effects of H_3_-receptor ligands on H_3_-receptor homodimerization in HEK 293 cells. HEK 293 cells expressing H_3_R-RLuc and H_3_R-YFP (200–500 fmol per mg protein; sub-maximal BRET) were incubated with isoproterenol (1 µM) or H_3_ ligands at fixed (100 nM) (**A**) or increasing (**B**) concentrations. (**C**) BRET kinetic analysis of ligand-induced BRET changes in living HEK 293 cells co-transfected with H_3_R-Rluc and H_3_R-YFP. (**D**) Membranes prepared from HEK 293 cells co-expressing H_3_R-RLuc and H_3_R-YFP were incubated with various nucleotides (100 µM). The results are representative of three or four experiments carried out in triplicate. Data are expressed as a percent of BRET signals in controls. * *p* < 0.05; ** *p* < 0.01; *** *p* < 0.001 versus control.

We investigated the effect of various H_3_R agonists and inverse agonists on constitutive BRET signals observed in living HEK (H_3_R) cells (Figure 3). All agonists, including histamine and standard H_3_R agonists (R)-α-methylhistamine [32] and imetit [48], significantly decreased (by ~10%) maximal BRET signals (Figure 3A). In contrast, all inverse agonists, including the standard compounds thioperamide [32], ciproxifan [49], and clobenpropit [50], significantly increased BRET signals (by ~7%) (Figure 3A). The protean agonist proxyfan [36,51] mimicked the effect of agonists and decreased BRET signals to the same extent. The β-adrenergic agonist isoproterenol did not significantly modify BRET signals (Figure 3A). Histamine and imetit decreased BRET in a dose-dependent manner, with similar maximal effects and EC_50_ values of 28 ± 2 and 4 ± 2 nM, respectively (Figure 3B), leading to relative potencies in the same range as those observed in H_3_R-mediated responses [48] (Figure 3B). The increase induced by the inverse agonist thioperamide was also concentration-dependent and saturable. It occurred with an EC_50_ value of 2 ± 1 nM (Figure 3B), in the same range as its inverse agonist potency on various H_3_R-mediated responses [41]. The BRET changes induced by imetit and thioperamide were time-dependent. They increased rapidly to reach a plateau within 10 min after drug exposure (Figure 3C). The BRET signals obtained without exposure to the ligands remained stable over 30 min (data not shown).

The addition of the stable GTP analog GTPγS (100 µM) mimicked the effect of inverse agonists and enhanced BRET signals by the same amplitude (Figure 3D). This effect was partially reproduced with GDP (100 µM), whereas ATP and CTP (100 µM) had no significant effect (Figure 3D). 

**Figure 4:**
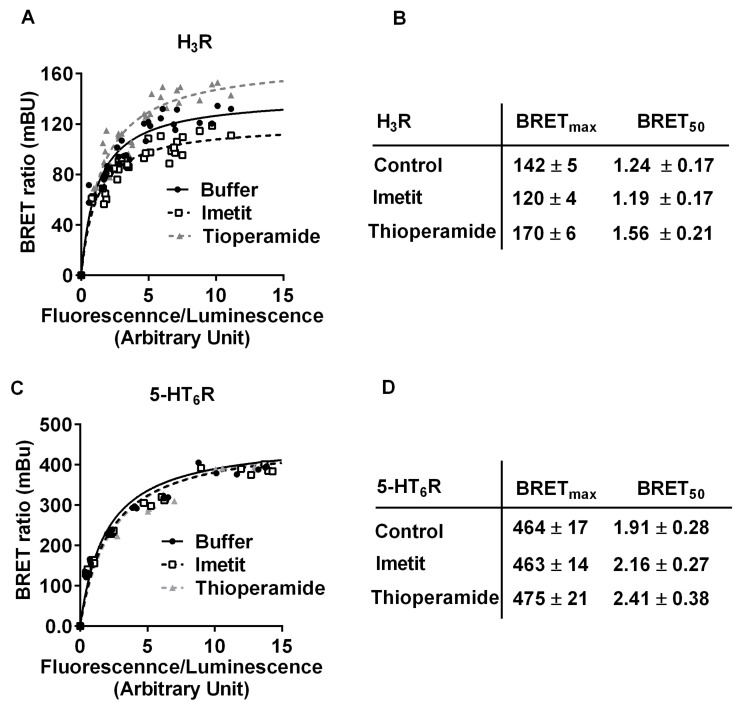
BRET saturation curves of homodimerization of H_3_R and 5-HT_6_R in HEK-293 cells. (**A**) BRET signals were performed on HEK 293 cells transiently transfected with a constant DNA amount of H_3_R-RLuc (**A**) or 5-HT_6_-RLuc (**C**) and increasing quantities of, respectively, H_3_R-YFP (**A**) or 5-HT_6_-YFP (**C**). BRET signals were determined in the absence (control) or presence of 100 nM of the H3R agonist (imetit) or 100 nM of the H_3_R inverse agonist (thioperamide). The curves were fitted using a non-linear regression equation assuming a single binding site (GrapPadPrism). The goodness of fit was given by r^2^ values that were very close to unity in each dose–response curve: r^2^ = 0.928, 0.926, and 0.928 for control, imetit, and thioperamide, respectively. Data were obtained from three separate experiments with triplicate determinations. (**B**,**D**) Parameters derived from BRET saturation curves of the homodimerization of H_3_R (**A**) or 5-HT_6_R (**B**) in the absence or presence of imetit or thioperamide. Data were analyzed by two-way ANOVA as follows: factor BRET level F(13, 42) = 195.5, *p* < 0.0001; factor treatment F(2, 42) = 104.1, *p* < 0.0001; and interaction F(26, 42) = 3.53, *p* = 0.0001.

The magnitude of the opposite effects of the agonist imetit and inverse agonist thioperamide was inversely related to BRET levels (Figure 4A,B). It was significantly higher at low BRET levels (20–40% of basal BRET_15–30_) than at higher BRET levels (~10% of basal BRET_75-max_). When BRET values were analyzed in the presence of imetit or thioperamide, the BRET_max_ values were significantly different from controls (*p* < 0.001), whereas the BRET_50_ values remained unchanged (BRET_50_ basal = 1.24 ± 0.017; BRET_50_ imetit = 1.19 ± 0.17, and BRET_50_ thioperamide = 1.56 ± 0.21). The H_3_R ligands did not significantly modify the BRET signals obtained after co-expression in the cells of the serotonin 5-HT_6_ receptors, 5-HT_6_R-RLuc and 5-HT_6_R-YFP (Figure 4C,D), indicating that changes induced by these ligands were selective of H_3_R dimers. 

### 2.4. Effects of H_3_R Ligands on BRET in Cultured Cortical Neurons

**Figure 5:**
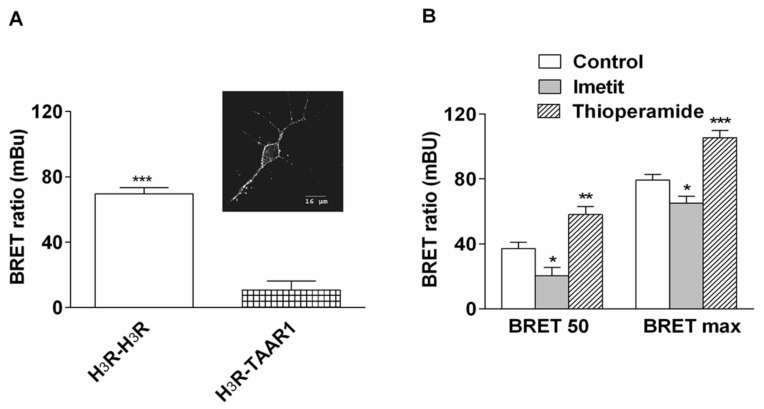
Effects of H_3_-receptor ligands on BRET signals in cultured cortical neurons. (**A**) Sub-cellular localization of H_3_R-YFP in rat cortical neurons, as observed by confocal microscopy two days after transfection. (**A**) Cortical neurons were transfected with H_3_R-RLuc and H_3_R-YFP or TAAR1-YFP as a control receptor in order to obtain sub-maximal BRET signals. A similar level of H_3_R-YFP and TAAR1-YFP expression was ensured by fluorescence analysis. Data are the means of 6 values from two independent experiments. *** *p* < 0.001 versus H_3_R-TAAR1 heterodimers. (**B**) Effects of ligands (100 nM) on H_3_R dimerization were evaluated at two different BRET levels. Means ± SEM of 18–24 values from 3–4 separate experiments. Two way ANOVA was performed as follows: factor BRET F(1, 99) = 152.2, *p* < 0.001; factor treatment F(2, 99) = 34.8, *p* < 0.001; and interaction F(2, 99) = 0.196, *p* = 0.8. Then, the effect of ligands was evaluated in each BRET group using a non-parametric Wilcoxon/Mann–Whitney test, * *p* < 0.05, ** *p* <0.01, *** *p* < 0.001 versus respective control.

BRET experiments were also conducted on living cortical neurons in primary culture (Figure 5). The sub-cellular localization of the fluorescent receptor expressed in neurons indicated that the H_3_R was predominantly found at the plasma membrane (Figure 5A). BRET signals were spontaneously generated in neurons after their co-transfection with H_3_R-YFP and H_3_R-RLuc. The specificity of this signal was illustrated by the absence of any significant energy transfer between H_3_R-RLuc and the control TAAR1 receptor (TAAR1-YFP) (Figure 5A). The constitutive BRET_max_ in neurons was about half that observed in HEK-293 cells (~70 versus ~140 mBU). Imetit and thioperamide (100 nM) did not modify the net BRET ratio when the donor H_3_R-RLuc was expressed alone but induced a significant decrease and increase, respectively, of the net BRET ratio obtained after co-expression of H_3_R-RLuc and H_3_R-YFP in cortical neurons (Figure 5B). The changes observed in neurons were much higher than those observed in HEK-293 cells. Thus, at half-maximal BRET of the controls, imetit decreased constitutive BRET signals by 45 ± 9%, whereas thioperamide increased them by 56 ± 13%. As observed in fibroblasts, the changes were inversely related to BRET levels. Interestingly, imetit still decreased constitutive BRET_max_ by 18 ± 4%, and thioperamide still increased BRET_max_ by 33 ± 8% (Figure 5B). 

### 2.5. Comparison of the Effects of H_3_R Ligands on H_3_R-Mediated BRET Signals and [^35^S]GTPγS Binding in HEK 293 Cells and Rat Cerebral Cortex

In order to further determine whether the changes of BRET induced by H_3_R ligands were related to their functional properties at the H_3_R, we compared the effects of the agonists histamine and imetit, the inverse agonists thioperamide and clobenpropit, and the protean agonist proxyfan on both [^35^S]GTPγS binding used as a functional response and BRET signals.

**Figure 6:**
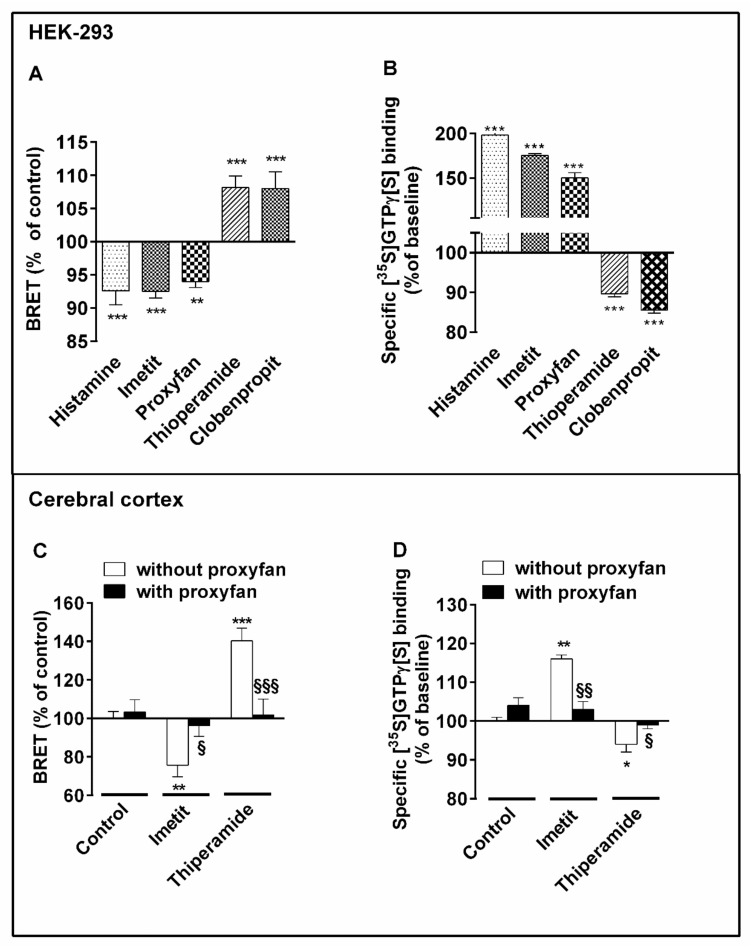
Effects of H_3_-receptor ligands on BRET signals and H_3_R-mediated [^35^S]GTPγS binding in HEK 293 cells and rat cerebral cortex. (**A**) HEK 293 cells expressing H_3_R-RLuc and H_3_R-YFP (200–500 fmol per mg protein; sub-maximal BRET) were incubated with the indicated H_3_R ligands (100 nM) ** *p* <0.01, *** *p* < 0.001 versus control (**B**) For [^35^S]GTPγS binding assays, membranes from HEK 293 cells expressing the H_3_R (200–500 fmol per mg protein) were incubated with H_3_ ligands at 100 nM. Means ± SEM of 8–16 values from two experiments. *** *p* < 0.001 versus control. (**C**) Cultured cortical neurons expressing H_3_R-RLuc and H_3_R-YFP were incubated with H_3_ ligands (100 nM) in the absence or presence of proxyfan (10 µM). Means ± SEM of 15–30 values from five separate experiments. ** *p* < 0.01; *** *p* < 0.001 versus control; § *p* < 0.05; §§§ *p* < 0.001 versus without proxyfan. (**D**) [^35^S]GTPγS binding assay was performed on membranes from the cerebral cortex of adult rats without or with H_3_ ligands at 100 nM. Means ± SEM of 8–16 values from two experiments. * *p* < 0.05; ** *p* < 0.01; versus control; § *p* < 0.05; §§ *p* < 0.05 versus without proxyfan.

In membranes from HEK 293 cells, histamine and imetit induced the expected decreases in BRET (Figure 6A) and increases in specific [^35^S]GTPγS binding (Figure 6B). In contrast, the inverse agonists thioperamide and clobenpropit significantly increased BRET (Figure 6A) and decreased [^35^S]GTPγS binding (Figure 6B), thereby confirming the existence of H_3_R constitutive activity in the system. The protean agonist proxyfan behaved as a partial agonist for both BRET and [^35^S]GTPγS binding, its effect reproducing that of histamine and imetit but with a lower magnitude (Figure 6A,B).

In rat cerebral cortex, imetit significantly decreased BRET signals in cortical neurons (Figure 6C) and increased [^35^S]GTPγS binding to membranes from adult rat cerebral cortex (Figure 6D). In contrast, thioperamide significantly increased BRET signals in neurons (Figure 6C) and decreased [^35^S]GTPγS binding to membranes by abrogating constitutive activity of the H_3_R (Figure 6D). In cultured neurons from the cerebral cortex, the protean agonist proxyfan had no effect alone on BRET signals but totally inhibited the opposite effects of imetit and thioperamide, indicating that it was acting as a neutral antagonist on BRET signals [51] (Figure 6C). Proxyfan also behaved as a neutral antagonist on [^35^S]GTPγS binding to adult membranes. Added alone, it had no effect, but it entirely blocked the opposite effects of imetit or thioperamide (Figure 6D). Since endogenous histamine levels in the medium of cortical neurons were not detectable, and proxyfan did not modify BRET signals when tested alone, it can be concluded that the effect of thioperamide resulted only from inhibition of H_3_R constitutive activity. 

## 3. Discussion

BRET analysis, co-immunoprecipitation studies, and Western blot experiments confirm that H_3_ receptors (as many, if not all GPCRs) exist under dimeric forms. In a previous report, H_3_R dimerization required cross-linking, making its physiological significance doubtful [43]. However, in the present study, spontaneous dimerization was clearly observed not only for recombinant H_3_Rs expressed in heterologous systems but also for recombinant H_3_Rs expressed in cerebral neurons as well as for native brain receptors. In addition to dimers, BRET and Western blot signals suggest that H_3_R also form higher oligomers. However, these oligomeric forms were hardly detected by co-immunoprecipitation, thereby suggesting that H_3_Rs predominantly exist as dimers. 

The physiological existence of “protean” agonism has been reported in cell lines and tissues [36,52]; this pharmacological concept is derived from constitutive activity and introduced by Kenakin on theoretical grounds [53]. We suggest that this protean agonism results from an equilibrium between inactive, ligand-directed active, and constitutively active conformations of the H_3_R [51]. The main finding of the present study is that BRET is an excellent biosensor that allows for biophysical evidence of these various allosteric H_3_R conformations.

Indeed, we show that spontaneous BRET signals correspond to constitutively active conformations. Minimal BRET signals correspond to agonist-induced conformations, whereas maximal BRET signals correspond to inactive conformations generated by inverse agonists. These BRET variations, therefore, unravel allosteric conformational changes of H_3_R dimers. Although the molecular nature of these changes remains unknown, they likely yield changes in the position or orientation of the Luc and YFP moieties [23,54,55,56], i.e., changes in the distance between the two BRET reporters. Both constitutive activity and agonists would promote an “opening” of the H_3_R dimer, enhancing the distance between protomers and, thereby, decreasing BRET signals. In contrast, the optimal BRET signals observed with inverse agonists indicate that the distance between the C terminal parts of the two protomers becomes minimal within inactive conformations of the receptor dimer. These BRET changes promoted by H_3_R ligands did not occur in a random fashion but paralleled the pharmacological profile of the receptor in a functional response such as [^35^S]GTPγS binding in both cells and neurons. Altogether, these data suggest that changes in BRET signals induced by H_3_R ligands reflect conformational changes linked to the activation process. We previously reported that H_3_R constitutive activity in cells and tissues required a level of expression higher than 80 fmol per mg protein in order to be detectable in various transductional responses [38,41]. The modulation of BRET signals induced by inverse agonists in neurons was detected at a density as low as ~20 fmol per mg protein, indicating that BRET is a very sensitive approach to detect the constitutive activity of the H_3_R. The BRET changes occurred with a time course consistent with the association kinetics of the ligands to the brain H_3_R [57,58]. Interestingly, GTPγS increased BRET signals to the same extent as inverse agonists, indicating that the same inactive conformations could be promoted not only via allosteric transconformations of constitutively active dimers by inverse agonists but also via the uncoupling of the same dimers from G proteins.

In agreement with previous studies on other GPCRs [25,59,60,61], changes in BRET signals induced by agonists or inverse agonists strongly suggest the existence of allosteric cross-talks between the two H_3_R protomers. In agreement, a previous study established that GPCRs of Class A, to which the H_3_R belongs [62], operate through the transactivation between the two protomers [8,26]. As reported for the GABAB receptor [63], maximal activation of dimers was ensured by agonist binding to a single protomer. Moreover, the dimer function was regulated by the activity state of the second protomer [26]. Interestingly, as also reported with Class C GPCRs [30,31], inverse agonist and agonist binding to a protomer facilitated and blunted, respectively, the activation of the adjacent protomer [26]. Other studies are also consistent with such an asymmetry between the two protomers in activated Class A GPCRs [1,64]. The increase and decrease in BRET signals that we observe with inverse agonists and agonists/constitutive activity, respectively, strongly suggest a similar scenario for H_3_R dimers. If confirmed by other approaches, our findings would, therefore, support an asymmetrical activation of H_3_R dimers, with distinct conformations and functions of the two protomers, the first one being activated by the agonist and the second one serving as a fine-tuner of the first. 

The interest in such a scenario is to reconcile or resolve some findings or questions raised by previous studies on GPCRs, including H_3_Rs. The negative cooperativity, repeatedly observed with H_3_R agonists in binding experiments with brain membranes and full or partial [^3^H]-agonists, was initially thought to reflect the existence of two distinct populations of sites with high and low affinity, respectively [57,65,66]. However, it was surprisingly maintained with recombinant receptors from various species [67,68,69,70] and was rather due to the fact that agonist binding to the second protomer blunted agonist binding to the first one. The positive cooperativity observed at recombinant or cerebral H_3_Rs with antagonists, then reclassified as inverse agonists, remained unexplained and likely reveals the facilitation of agonist binding to the first protomer induced by the binding of inverse agonists to the second protomer [65,69]. 

Such a scenario, based on the transactivation of two protomers, may also lead us to reconsider the existence that we have proposed—a simple competition between ligand-directed and constitutively active conformations of the H_3_R for G proteins. The inverse relationship between the efficacy of agonists and the level of constitutive activity that we observed in both native and recombinant H_3_Rs [41,71] may not only reflect such a competition, as has been assumed so far [51], but also the allosteric interactions between the two protomers. Indeed, this apparent inverse relationship between agonism and constitutive activity may result as well from the fact, as suggested by BRET changes, that agonism at one promoter is progressively blunted when the constitutive activity of the second promoter increases [26]. Such an inverse relationship likely exists between endogenous histamine acting as the physiological H_3_R agonist and H_3_R constitutive activity. In fact, after the evidence for a physiological role of H_3_R constitutive activity in vivo, the role of endogenous histamine in the activation of brain H_3_Rs was questioned [36]. We suggested that the constitutive activity of H_3_ autoreceptors located on the cell bodies of histaminergic neurons was higher than that of H_3_ autoreceptors located on nerve terminals because the effects of inverse agonists on histamine neuron activity in vivo are much higher than their effects on histamine release in vitro [41]. Considering H_3_Rs as asymmetrical dimers may help us to understand the functional role of histamine, which would be predominant at presynaptic autoreceptors with low constitutive activity of the second protomer but largely blunted at somato-dendritic autoreceptors by the much higher constitutive activity of this second protomer.

The regulation of BRET signals by agonists, inverse agonists, and GTPγS definitely show that the minimal functional signaling units of the H_3_R are not monomers but dimers coupled to G proteins. However, although their formation by dissociation of dimers during the experiments cannot be entirely ruled out, our findings from Western blot strongly suggest that H_3_R monomers co-exist with dimers and/or oligomers in the brain and cells. Moreover, if various studies suggest that dimerization of GPCRs is a prerequisite for G-protein activation [72,73,74,75], a single molecule of rhodopsin [76] or β2-adrenergic receptor [77] can efficiently activate G proteins when reconstituted in a nanodisc. However, whether such a coupling of a single protomer occurs in vivo is unknown, and the absence of the second regulatory protomer would be expected to induce an over-activation of the monomer, leading to its rapid internalization. Whatever the presence and function of H_3_R monomers in the brain, our findings show that they cannot be generated by the dissociation of dimers [22,23,54,78], even though H_3_R agonists tended to promote the “opening” of the dimer, i.e., decreased BRET signals. Firstly, the unchanged relative affinity between the protomers (unchanged BRET 50 values), as well as the changes of BRET_max_ in cells, suggest that BRET changes promoted by the H_3_R ligands reflect transconformations. Secondly, agonists and constitutive activity only partially decrease but do not abolish BRET signals. Finally, using Western blot analysis, we were not able to detect the dissociation of H_3_R dimers after treatment with agonists (Figure S4). Altogether, our findings are consistent with the consensus that ligand-induced changes in BRET reflect conformational changes within pre-existing dimers and not changes in the equilibrium between monomers and dimers [16,18,25]. 

The conformational changes observed in intact living cells resulted in a modest (10% to 20%) increase or decrease in BRET signals. However, these changes were highly reproducible and were in the same range as those observed for other Class A GPCRs [79,80]. Moreover, the changes (by 30% to 40%) induced in neurons by H_3_R ligands were stronger than in fibroblasts. This observation may be attributable to a higher coupling efficacy of the receptor and/or to increased receptor-G protein stoichiometry [81] in neurons than in non-neuronal cells. This better coupling would be accompanied by higher constitutive activity in neurons than in fibroblasts. In agreement, the increase in BRET signals induced by the standard inverse agonist thioperamide was higher in neurons than in cells (+45% versus +21%). Additionally, consistent with higher H_3_R constitutive activity in neurons than in cells, the protean agonist proxyfan behaved as an agonist in cells but became a neutral antagonist in neurons, not only upon [^35^S]GTPγS binding but also upon BRET signaling. Moreover, the stronger effect of H_3_R ligands observed in neurons may also result from more favorable orientations of the two protomers within H_3_R dimers to detect ligand-induced conformational changes. 

Both the high density of H_3_Rs at the plasma membrane of cells and neurons and the high dimerization observed within the ER show that H_3_R dimers are formed at the early stages of receptor biosynthesis and trafficking. It has been proposed that this early dimerization occurs to warrant that only properly folded receptors reach their site of action [16]. The extent of the surface expression of GPCRs would be, therefore, dependent on their ability to form dimers and/or oligomers at the pro-receptor stage. The upregulation of GPCRs, including the H_3_R [37,40,82], induced by sustained treatment with inverse agonists would then result from a decreased constitutive desensitization of the receptor [83,84] and a stabilization of the membrane dimers by inverse agonists [26]. The capacity of H_3_R protomers to dimerize was, however, not dependent on their intracellular or membrane localization per se because the H_3_R did not dimerize with the TAAR1 receptor, which is mainly intracellular [85], and hardly dimerized with the D_3_ receptor, which is predominantly found at the cell membrane [86]. 

In conclusion, dimeric forms of H_3_Rs are regulated by agonists and inverse agonists. All our data are consistent with a model in which transactivation between the two protomers of pre-existing dimers underlies H_3_R activation. The present data also indicate that major regulations induced by the constitutive activity of H_3_Rs in neurons may occur at three distinct levels: the drug-intrinsic property [51], the receptor itself (dimers), and its associated biological responses [38,40,41]. H_3_R inverse agonists have entered clinical trials for the treatment of arousal, cognitive, and food intake disorders [35,36]; functional dimers represent, with H_3_R functional isoforms and species differences, an additional level of complexity that may influence their therapeutic effects.

## 4. Materials and Methods

### 4.1. Plasmid Constructs

Rat H_3_ receptor cDNA, corresponding to the long isoform of the receptor (H_3_(445)R) without its stop codon, was amplified by PCR. The product was sub-cloned in a frame into the NheI/BglII site of the pEYFP-N1 vector (Clontech, San Jose, CA, USA) encoding the YFP variant of the green fluorescent protein and into the NheI site of the pRL-CMV-RLuc vector (Promega, Madison, WI, USA) to generate H_3_R-YFP and H_3_R-RLuc fusion proteins, respectively. Renilla Luciferase (RLuc) and YFP were inserted at the C-terminal end of the receptor. All constructs were checked using direct DNA sequencing.

### 4.2. Animals

All animal protocols were carried out according to French Government animal experiment regulations and were approved by Animal Ethical Committees (Comité d’Ethique pour l’Expérimentation Animale Orléans CE03) and accredited by the French Ministry of Education and Research (MESR) under national authorization number #C45231412.

### 4.3. Cell Cultures and Transfections

Cortical neurons were prepared from 18-day-old embryos of male Wistar rats (Janvier, Le Genest-St-Isle, France). Neurons were plated on polyornithin-coated plastic culture dishes for BRET experiments and glass coverslips for immunofluorescence (50,000 cells per cm^2^). Cultures were grown in neurobasal medium (Invitrogen, Waltham, MA, USA) supplemented with B27 (Invitrogen) and 2 mM L-glutamine. Neurons (8 days in vitro) were transfected with lipofectamine 2000 (InvitrogenTM, Life technologies, Carlsbad, CA, USA). HEK 293 cells (European Collection of Cell Cultures, ECACC Ref 85120602 (Sigma-Aldrich, St Louis, MO, USA) were transfected using the calcium phosphate precipitation method.

### 4.4. Membrane Preparations

Forty-eight hours after transfection, membranes from HEK 293 cells were prepared and suspended in the appropriate ice-cold buffer for [^125^I]iodoproxyfan binding assays (Na_2_HPO_4_/KH_2_PO_4_ 50 mM, pH 7.4), [^35^S]GTPγS-binding assays (Tris-HCl 50 mM, pH 7.4), Western blot analysis (Tris-HCl 50 mM, pH 7.4, 5 mM EDTA with protease cocktail inhibitor), and BRET assays (Tris-HCl 50 mM, pH 7.4). Crude membranes from rat cerebral cortex, cerebellum, and hypothalamus were prepared [65] and suspended in ice-cold Western blot buffer.

### 4.5. Immunoprecipitation Assays 

HEK 293 cells were co-transfected with C-terminal YFP-fused and N-terminal Xpress-tagged H_3_Rs. Forty-eight hours after transfection, cells were washed with ice-cold PBS and lysed in buffer containing 50 mM Tris pH 7.5, 150 mM NaCl, 10 mM EDTA, and 0.5% Triton X-100 plus protease cocktail inhibitor on ice for 10 min. The lysates were then centrifuged at 10,000× *g* for 10 min. The supernatants were incubated with protein A-sepharose (GE Healthcare, Chalfont St. Giles, UK) and anti-GFP antibodies (BD Bioscience Clontech, San Jose, CA, USA) overnight at 4 °C. The beads were washed five times with lysis buffer and resuspended in 4-fold concentrated Laemmli buffer (200 mM Tris-HCl pH 6.8, 4% SDS, 40% glycerol, 0.02% bromophenol, βME 0.5 M). Non-specific background was determined by the transfection of N-terminal Xpress-tagged H_3_Rs without H_3_R-YFP.

### 4.6. Western Blots

The cell lysates, immunoprecipitates, or membranes from various rat or mouse brain regions were separated by electrophoresis on SDS/PAGE (8% or 10% gels) or NuPAGE Tris-Acetate 3–8% or 7% precast gels (Thermo Fisher Scientific Inc,Rockford, IL, USA). under reducing conditions and transferred on polyvinylidene fluoride (PVDF) membranes (GE Healthcare Life Sciences, Chalfont St. Giles, UK). Blots containing Xpress or YFP-tagged receptors were probed with a mouse anti-Xpress antibody (1:1000, Invitrogen) or a rabbit anti-BD living colors full-length polyclonal antibody (1:3000, BD Biosciences). Immunoblots were also probed with a rabbit anti-H_3_R polyclonal antibody (1:1000, Livespan Biosciences). Horseradish–peroxidase-conjugated goat anti-mouse or anti-rabbit antibodies (1:33,000 dilutions) were used as secondary antibodies (Promega, Madison, WI, USA). Immunoreactive bands were detected using the Pico or Dura detection kit (Thermo Fisher Scientific Inc (Rockford, IL, USA).

### 4.7. Binding Assays

For H_3_R radioligand binding assays, membranes were incubated with [^125^I]iodoproxyfan as described [65]. For [^35^S]GTPγS-binding assays, membranes were pretreated and incubated with 0.1 nM [^35^S]GTPγS and the H_3_ ligands, as described [38]. Statistical evaluation of the results was performed using one-way ANOVA followed by Student’s Newman-Keuls posthoc test.

### 4.8. Cell Fractionation Studies

Forty-eight hours after transfection, HEK 293 cells were washed with PBS, scraped off, and lysed with cold hypotonic lysis buffer containing 20 mM HEPES, pH 7.4, 2 mM EDTA, 2 mM EGTA, 6 mM MgCl_2_, 1 mM PMSF, and protease cocktail inhibitor. Cell suspensions were homogenized and lysates centrifuged at 1000× *g* for 5 min. The supernatant was collected, and sucrose was added to obtain a final concentration of 0.2 M. Cell lysates were applied to the top of a discontinuous sucrose step gradient (5 mL per step), made at 0.5, 0.9, 1.2, 1.35, 1.5, and 2.0 M sucrose in lysis buffer. The samples were centrifuged (27,000× *g* for 16 h). Fractions were then submitted to fluorescence/luminescence and BRET analysis. Identification of plasma membranes and ER-enriched fractions was achieved by Western blot using rabbit polyclonal anti-Na+/K+-ATPase (Sigma Aldrich, St Louis, MO, USA) and anti-calnexin (Santa Cruz Biotechnology, Santa Cruz, CA, USA) antibodies, respectively.

### 4.9. Luminescence, Fluorescence, and BRET Assays

Forty-eight hours after transfection, HEK 293 cells or cultured cortical neurons were detached with versene (Invitrogen) and resuspended in HBSS saline buffer (Invitrogen). Intact cells or membranes were distributed in 96-well microplates (Optiplate, Perkin Elmer, Waltham, MA, USA) and incubated for 15 min at 25 °C in the absence or presence of the indicated ligands. Coelenterazine H substrate (Interchim) was added at a final concentration of 5 µM, and reading was performed with a Mithras LB 940 multireader (Berthold, Bad Widbad, Germany), which allows the sequential integration of luminescence signals detected with two filter settings (RLuc filter, 485 ± 10 nm; YFP filter, 530 ± 12 nm). Emission signals at 530 nm were divided by emission signals at 485 nm. The difference between this emission ratio, obtained with co-transfected RLuc and YFP fusion proteins, and that obtained with the RLuc fusion protein alone is defined as the BRET ratio. The results are expressed in milliBRET units (mBU, with 1 mBU corresponding to the BRET ratio values multiplied by 1000). BRET_max_ is the maximal BRET signal obtained in milliBRET units, and BRET50 represents the ratio of acceptor and donor receptors (acceptor/donor), yielding 50% of the maximum BRET signal. Statistical evaluation of the results was performed using one-way ANOVA followed by the Newman-Keuls posthoc test or two-way ANOVA followed by a non-parametric Wilcoxon/Mann–Whitney test.

Fluorescence was measured in black 96-well plates (Optiplate, Perkin Elmer) using the Mithras LB 940 reader (Berthold) with an excitation filter at 480 nm and an emission filter at 510 nm (gain, 1; photomultiplicator tube, 2000 V; time, 1.0 s). Total luminescence of cells was determined in white 96-well plates (Otiplate, Perkin Elmer). Background luminescence and fluorescence determined in wells containing untransfected cells were subtracted.

### 4.10. Fluorescence Microscopy

Neurons cultured for 8 days were transfected using lipofectamine 2000 with the plasmid encoding H_3_R-YFP. Cells were fixed with 4% paraformaldehyde two days after transfection. Images were acquired using a TCS-SP2 confocal laser scanning microscope (Leica Biosystems, Nanterre, France).

### 4.11. Analysis of Data

For BRET saturation or modulation, the total curves were analyzed with an iterative least-squares method derived from that of Parker and Waud [87]. Computer analysis was performed by non-linear regression using a one-site cooperative model. The method provided estimates for EC_50_ values, BRET_max_ and BRET_50_ values, and their SEMs. Statistical evaluation of the results was performed using one-way ANOVA, followed by Student’s Newman-Keuls posthoc test or by two-way ANOVA.

### 4.12. Radiochemicals and Drugs

[^125^I]Iodoproxyfan (2000 Ci/mmol) was prepared as described [88,89]. [^35^S]GTPγS (1250 Ci/mmol) was from Perkin Elmer Life Sciences (Boston, MA, USA). Clobenpropit was from Tocris (Bristol, UK). R-α-methylhistamine, ciproxifan, and proxyfan were provided by W. Schunack (Freie Universität Berlin, Berlin, Germany). Histamine, imetit, thioperamide, and isoproterenol were purchased from Sigma-Aldrich (Saint Quentin Fallavier, France). All other chemicals were from commercial sources and were of the highest purity available. Interventionary studies involving animals or humans and other studies that require ethical approval must list the authority that provides the approval and the corresponding ethical approval code.

## Data Availability

All data included in the study are presented in the manuscript. The corresponding author bears as guarantor for data validation.

## Supplementary Materials

The following are available online at https://www.mdpi.com/article/10.3390/ijms221910638/s1, Figure S1: Deglycosylation of H_3_R-YFP with endoglycosidase H (Endo H) and peptide-N-glycosidase (PNGase F); Figure S2: Characterization of the H3 receptor antibody by immunolabeling in Cos7-transfected cells; Figure S3: Influence of H_3_R receptor density on BRET signals; Figure S4: Analysis of ligand-promoted changes in dimerization states.
