# Supplementary material for: BRET Analysis of GPCR Dimers in Neurons and Non-Neuronal Cells: Evidence for Inactive, Agonist, and Constitutive Conformations
Source: Int J Mol Sci. 2021 Sep 30;22(19):10638. doi: 10.3390/ijms221910638 (PMC8508734; doi:10.3390/ijms221910638)
Supplement: Supplementary file 1 [file ijms-22-10638-s001.zip › ijms-1387994-supplementary.pdf]

SUPPORTING INFORMATION

El Khamlichi et al.

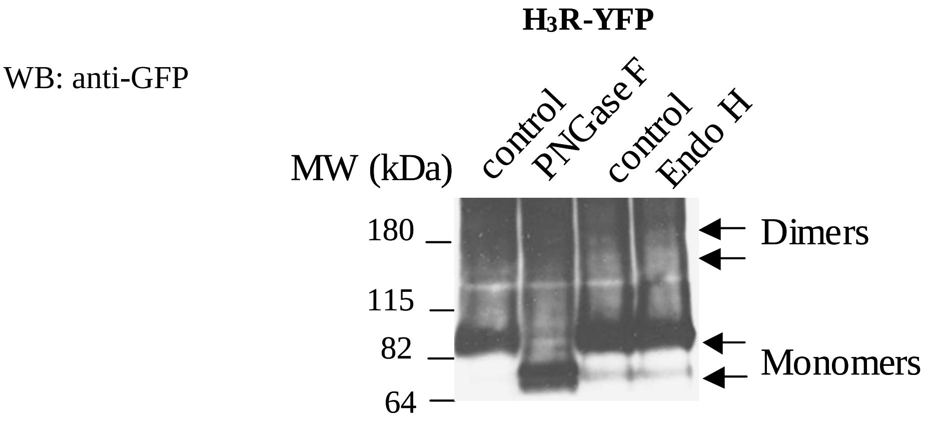

Figure S1: **Deglycosylation of H_3R-YFP with endoglycosidase H (Endo H) and Peptide-N-glycosidase (PNGase F).** Membranes of HEK-293 cells stably transfected with the H_3R-YFP were incubated in Buffer A (50mM sodium citrate, pH 5.5, 1% Triton, cocktail of protease inhibitor) or in Buffer B (50mM sodium citrate, pH 7.5, 1% Triton, cocktail of protease inhibitor) for Endo H or PNGase F treatment respectively for 2 hours at 37°C. The reaction was stopped by adding LDS sample buffer (Invitrogen). Samples were separated using pre-cast 3-8% Tris-acetate NuPAGE gels (Invitrogen) and analysed by immunoblotting with the anti-BD living colors antibody (BD Biosciences).

The 90 kDa monomers and the 180kDa dimers species were resistant to the treatment with EndoH. Therefore, they probably correspond to the mature monomeric and dimeric forms containing complex types of N-linked glycans. Treatment of membranes with PNGase F reduced the 90 kDa band to about 75 kDa. Dimers that appear as a smear at around 180 kDa seem to be reduced to lower species after PNGase F treatment. This allows us to suggest that the 75kDa species represent the non-glycosylated monomeric forms of the recombinant H_3R-YFP.

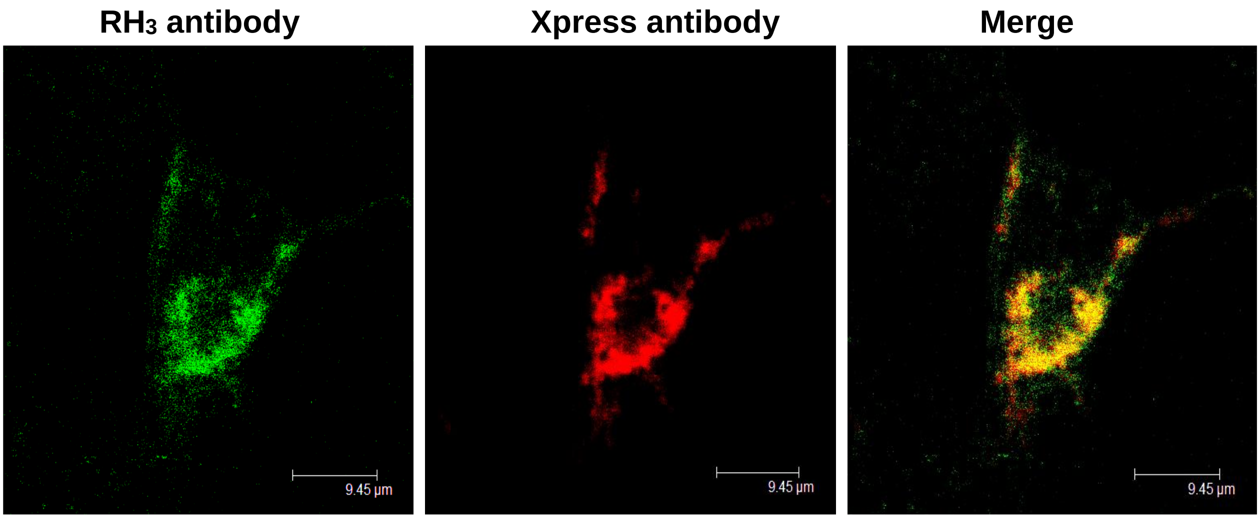

Figure S2: **Characterization of the H_3 receptor antibody by immunolabelling in Cos7 transfected cells.** COS-7 cells maintained in culture were detached using versene, plated on glass microscope coverslips and left to grow at 37°C for 24h. COS-7 cells were transfected using lipofectamine 2000 (Invitrogen) with the pcDNA3.1/His vector (Invitrogen) or the pcDNA3.1 (Xpress-H_3 receptor) vector and further incubated at 37°C for 48 h in a humidified atmosphere containing 5% CO_2 in air. Cells were washed with 0.1 M phosphate buffered saline (PBS, pH 7.2-7.4), fixed in a solution containing 3% paraformaldehyde (PFA) in PBS for 40 min at room temperature (RT). After three washes in PBS, cells were incubated for 10 min with NH_4Cl (50 mM), then permeabilized with 0.5% X100 Triton in PBS for 1 min. After washing in PBS, cells were incubated in 20 % normal goat serum for 1h at RT. The normal goat serum at 10 % in PBS was then used for the dilutions of primary and secondary antisera and the different washes. Cells were incubated with the monoclonal mouse anti-Xpress antibody (Invitrogen) at 1:200 for 1h at RT. After three washes, cells were then incubated overnight at 4°C with the polyclonal rabbit anti-H_3 receptor antibody (Lifespan Biosciences) at 1:200. After three washes, cells were incubated with a donkey anti-mouse IgG conjugated to Cy3 (FluoProbes) for 1h at RT and finally, after three additional washes, incubated with a goat anti-rabbit IgG conjugated to Alexa 488 (FluoProbes) at 1:200 for 30 min at RT. After rinsing in PBS (3 x 10 min), cells were mounted on glass slides with Vectashield (Vector Laboratories). When immunocytochemical labelling was carried out on cells by omitting the primary or secondary antibodies, no cellular labelling was observed. Images were acquired using a Leica TCS- SP2 confocal laser scanning microcope. No fluorescent signal was detected in cells transfected with the control vector (pcDNA3.1). The labelling obtained with the H_3 receptor antibody totally colocalized with the labelling obtained with the Xpress antibody.

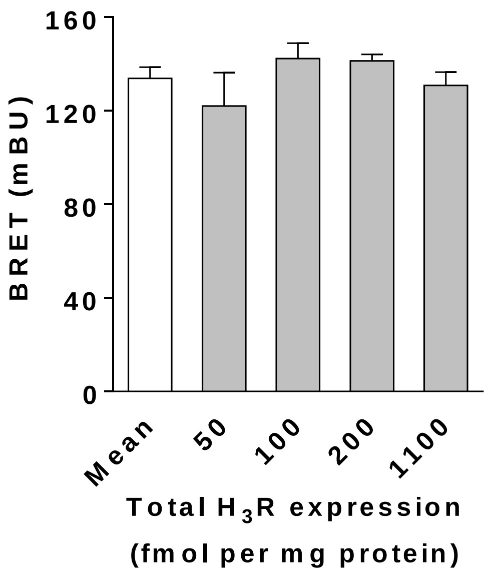

Figure S3: **Influence of H_3R receptors density on BRET signal.** Although the experiments were performed at receptor expression levels similar to those observed in native tissues, additional experiments were carried out to exclude the possibility that the BRET signal observed could result from overexpression in heterologous system. HEK-293 cells were transfecting with various concentrations of H_3R-YFP and H_3R-RLuc plasmids in keeping the ratio H_3-YFP/ H_3-Rluc constant (around ~ 2) to ensure a $BRET_{max}$ signal. The density of total H_3R was determined in the same cells by measuring the [^{125}I]iodoproxyfan binding. BRET signal remained constant over a wide range of H_3R density (from 50 fmol to 1 pmol per mg protein). Means ± S.E.M. of 8 determinations from 2 separate experiments.

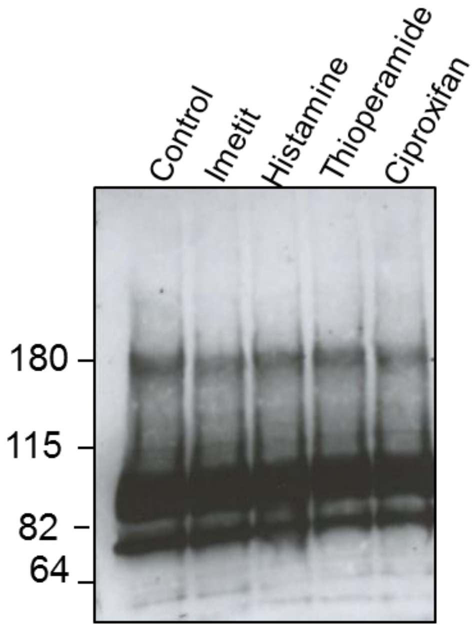

Figure S4: Analysis of ligand-promoted changes in dimerization states. HEK-293 cells stably expressing H_3R-YFP protein fusion were incubated with H_3R ligands (imetit, histamine as agonists and thioperamide, ciproxifan as inverse agonists) at fixed concentration (100 nM) for 30 minutes at 25°C. After membrane preparations, Samples were separated using pre-cast 3-8% Tris-acetate NuPAGE gels (Invitrogen) analysed by immunoblotting with an anti-GFP antibody. Immunoreactive bands corresponding to putative monomeric (75 and 90kDa) and dimeric (180 kDa) forms can be detected.
